# High-resolution characterisation of ESBL/pAmpC-producing *Escherichia coli* isolated from the broiler production pyramid

**DOI:** 10.1038/s41598-020-68036-9

**Published:** 2020-07-07

**Authors:** Ilias Apostolakos, Claudia Feudi, Inga Eichhorn, Nicola Palmieri, Luca Fasolato, Stefan Schwarz, Alessandra Piccirillo

**Affiliations:** 1 Department of Comparative Biomedicine and Food Science, University of Padua, 35020 Padua, Italy; 2 Institute of Microbiology and Epizootics, Centre for Infection Medicine, Department of Veterinary Medicine, Freie Universität Berlin, 14163 Berlin, Germany; 3 Department for Farm Animals and Veterinary Public Health, University Clinic for Poultry and Fish Medicine, University of Veterinary Medicine, 1210 Vienna, Austria

**Keywords:** Microbiology, Molecular biology

## Abstract

The presence of extended-spectrum β-lactamase (ESBL) or plasmid-mediated AmpC β-lactamase (pAmpC)-producing *Escherichia coli* (ESBL/pAmpC-EC) in livestock is a public health risk given the likelihood of their transmission to humans via the food chain. We conducted whole genome sequencing on 100 ESBL/pAmpC-EC isolated from the broiler production to explore their resistance and virulence gene repertoire, characterise their plasmids and identify transmission events derived from their phylogeny. Sequenced isolates carried resistance genes to four antimicrobial classes in addition to cephalosporins. Virulence gene analysis assigned the majority of ESBL/pAmpC-EC to defined pathotypes. In the complex genetic background of ESBL/pAmpC-EC, clusters of closely related isolates from various production stages were identified and indicated clonal transmission. Phylogenetic comparison with publicly available genomes suggested that previously uncommon ESBL/pAmpC-EC lineages could emerge in poultry, while others might contribute to the maintenance and dissemination of ESBL/pAmpC genes in broilers. The majority of isolates from diverse *E. coli* lineages shared four dominant plasmids (IncK2, IncI1, IncX3 and IncFIB/FII) with identical ESBL/pAmpC gene insertion sites. These plasmids have been previously reported in diverse hosts, including humans. Our findings underline the importance of specific plasmid groups in the dissemination of cephalosporin resistance genes within the broiler industry and across different reservoirs.

## Introduction

Third-generation-cephalosporins (3GCs) are critically important antimicrobial agents in human medicine as they are amongst the few available treatment options in infections caused by multi-drug resistant *Enterobacteriaceae*^1^. Their role in the treatment of food-producing animal diseases (e.g. respiratory infections in ruminants, foot rot and mastitis in cattle) is as well considered as crucial^2^. To preserve the efficacy of 3GCs, their administration in poultry within the European Union is not authorised^3^ but there is evidence of off-label use in hatcheries, either *in ovo* or subcutaneously in day-old chicks, to prevent early mortality due to *Escherichia coli* infections^4^. Nevertheless, several studies suggested that the broiler production system acts as a reservoir of 3GC-resistant bacteria, such as extended-spectrum β-lactamase (ESBL) or plasmid-encoded AmpC β-lactamase (pAmpC)-producing *E. coli* (ESBL/pAmpC-EC), given that their prevalence is higher in the broiler production industry compared to other animal sectors^5^. Further, presence of ESBL/pAmpC genes on highly transmissible plasmids, which enables their dissemination in diverse reservoirs^6^, initiated a debate on the exposure risk for consumers via the food chain^7,8^. However, the connection between human exposure and presence of ESBL/pAmpC-EC in broilers was supported by circumstantial evidences showing the presence of identical ESBL/pAmpC genes in closely related *E. coli* of both hosts inferred by low-resolution typing methods^9,10^. The importance of high-resolution, whole genome sequencing (WGS) studies in informing and reducing the uncertainty of quantitative microbial risk assessment (QMRA) models employed in the risk analysis of foodborne antimicrobial resistance was recently stressed^11,12^. Such an assessment for the broiler chain would require the thorough characterisation of ESBL/pAmpC-EC and their transmission patterns across the production pyramid^5^.

In a recent study, we detected a high prevalence of ESBL/pAmpC-EC in an integrated broiler production chain^13^. Here, we proceeded with the WGS-assisted characterisation of a selection of ESBL/pAmpC-EC isolates collected within the formerly published framework. The main aim of the study was to generate high-resolution data on ESBL/pAmpC-EC, advance our understanding on their characteristics and epidemiology in the broiler production and thus provide valuable information to (parts of) QMRA efforts. Our specific objectives were (i) to fully explore the antimicrobial resistance (AMR) and virulence profiles of the isolates (crucial for hazard identification) (ii) to characterise ESBL/pAmpC gene-carrying plasmids, study their epidemiology within the broiler production chain and assess their relevance in other reservoirs (exposure assessment), (iii) to infer the phylogeny of ESBL/pAmpC-EC and identify transmission events within the broiler production (exposure assessment), and (iv) set the isolates of this collection in context with other publicly available genomes and thereby assess their presence in other hosts.

## Results and discussion

### Phylogenetic analysis

**Figure 1:**
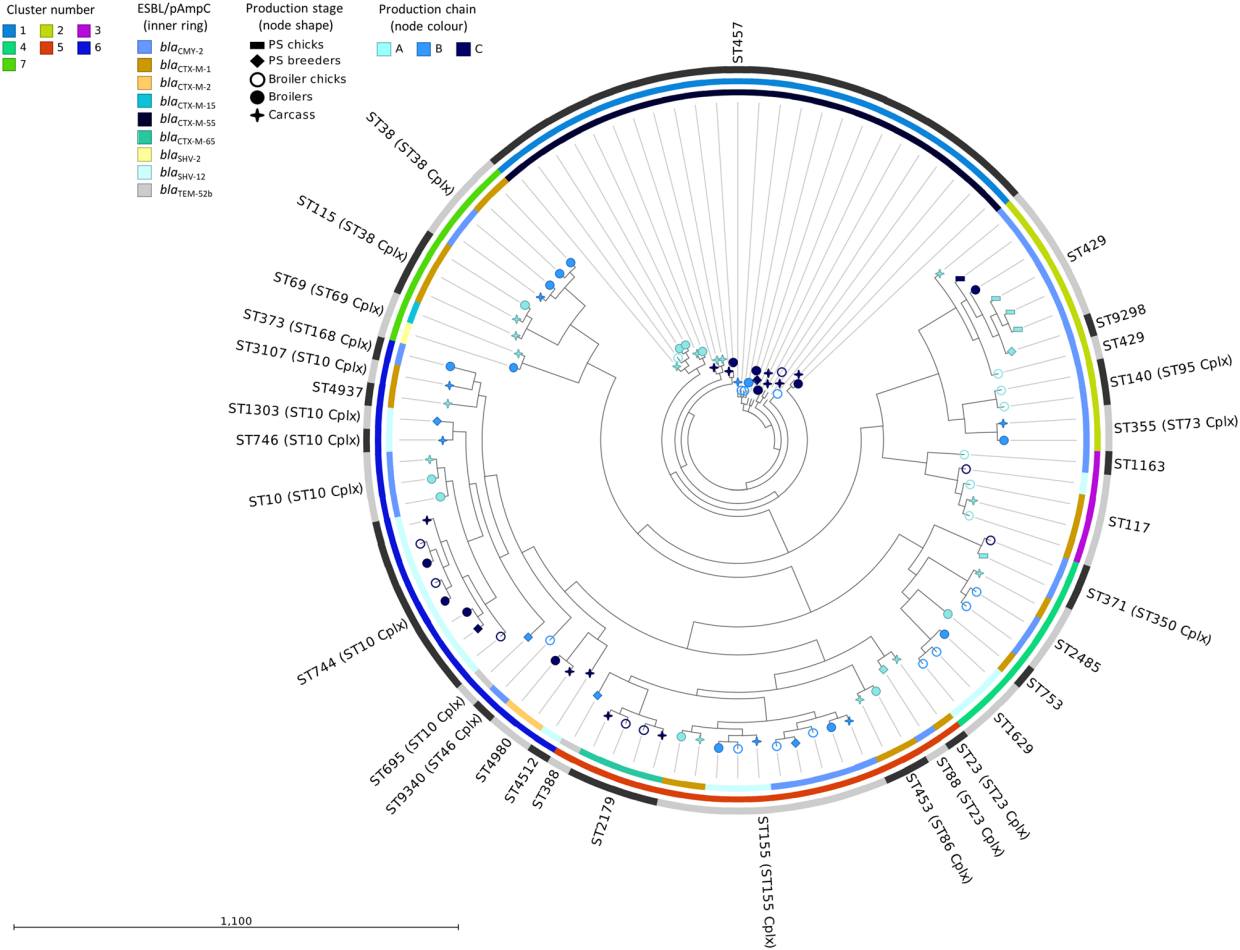
Phylogenetic analysis of the 100 sequenced isolates. The SNP tree was created with CSI phylogeny. Nodes shape and colour indicate the broiler production stage and production chain, respectively. The inner ring specifies the ESBL/pAmpC resistance genes identified. The middle ring indicates hierBAPS clusters. The outer ring with alternating dark and light grey segments, marks different STs. Scale bar refers to the branch lengths, which are measured in the number of substitutions per site.

In silico typing revealed a complex genetic background in the 100 sequenced *E. coli* isolates by assignment to 31 unique Sequence Types (STs) (Fig. 1). Two novel STs were identified with one representing isolate each. The ST9298 isolate was obtained from a breeder and is closely related to ST429 as they differ by only one SNP in the *icd* allele (${\text{T109A}}, {icd}13\rightarrow{icd}1072$). The ST9340 isolate was recovered from a day-old broiler chick and is closely related to ST46 of the ST Complex (Cplx) ST10 (${\text{C336T}}, {gyrB}1\rightarrow{gyrB}812$). Overall, ST457 and ST155 (comprising 25% and 10% of all isolates, respectively) were the most dominant STs followed by ST744 (ST10 Cplx; 8%) and ST429 (7%) (Table 1). The majority of the remaining isolates were assigned to various STs, represented by less than three isolates each, while ST38 (ST38 Cplx), ST117 and ST2179 were represented by four isolates each.

Genomes were mapped against the reference genome of *E. coli* strain MRY15-131 (Accession no. NZ_AP017620.1). All isolates covered at least 66.3% of the reference genome sequence resulting in a core genome alignment of 3.34 Mbp and 148,830 informative single nucleotide polymorphisms (SNPs) (File S2). Cluster analysis with hierBAPS revealed the presence of seven clusters. The examined isolates clustered primarily in accordance to their ST and subsequently to their ST complex (Fig. 1). Each of the four most dominant STs (ST457, ST155, ST744 and ST429/ST9298) was assigned to an independent cluster. For each of these STs, a separate in-depth SNP analysis was conducted with suitable reference genomes (File S2) to identify clonal transmission events within the studied broiler production pyramid (Fig. S1) and gain insights from the comparative genomic analysis with publicly available genomes (Fig. S2).

Among all isolates, ST457 was the most widespread ST (n = 25 isolates), representing isolates from all chains and stages of the production pyramid. All ST457 isolates harboured *bla*_CTX-M-55_ and exclusively formed Cluster 1 (Fig. 1). In-depth SNP analysis distinguished two clusters, Cluster 1 and Cluster 2, among the ST457 isolates, with a median of pairwise differences (MPD) among all isolates at 3,855 SNPs (Fig. S1a). The cluster 1 (n = 19 isolates) was formed by closely related isolates (MPD 13 SNPs), retrieved from various stages of all three production chains, whereas Cluster 2 (n = 6 isolates) consisted of closely related isolates of chain A, including broiler chicks, fattened broilers and carcasses (MPD 19 SNPs) (Fig. S1a). Given that in previous reports epidemiologically linked isolates had 0–23 SNPs^14^, our results indicate a clonal expansion of ST457 in the majority of the production chains within the studied broiler production system. ST457 is a rare ST, first reported in the United Kingdom as a nosocomial isolate obtained from an urine sample^15^. A *bla*_KPC-3_-*bla*_CTX-M-55_-carrying *E. coli* isolated from a human bloodstream infection in Italy^16^ was amongst the few recently reported ST457 isolates. An additional study conducted in the United States, showed the concurrent presence of *mcr-1* and *bla*_CTX-M-55_ on the same plasmid carried by an *E. coli* ST457 obtained from a human urinary tract infection^17^. Fifteen out of 22 ST457 genomes at Enterobase harboured ESBL/pAmpC genes, but none of them carried *bla*_CTX-M-55_ or was isolated from poultry. In the SNP analysis, six of our ST457 isolates clustered closely with two human *bla*_CTX-M-1_ carrying isolates from the Netherlands (unpublished) and one *bla*_CMY-2_-carrying isolate from Germany^14^ (Cluster 3, Fig. S2a), and these nine isolates had a MPD of 142 SNPs. In contrast, the remaining ST457 isolates clustered more distantly (MPD 638 SNPs) with livestock isolates (Fig. S2a).

In the overall phylogenetic analysis, Cluster 5, the second most diverse cluster in terms of varying ST content after Cluster 6 (Fig. 1), was mainly represented by ST155 (n = 10 isolates). Isolates of ST155 formed three clusters according to their ESBL/pAmpC gene content (*i.e. bla*_CMY-2_, *bla*_CTX-M-1_ and *bla*_SHV-12_) and the within-cluster SNP differences (MPD 5–11 SNPs) suggested clonal dissemination (Fig. S1b). SNP analysis including the respective Enterobase genomes showed that ESBL/pAmpC isolates of ST155 are common in poultry^8,14^ (n = 22/35 isolates were of poultry origin). The isolates of this study were grouped in a large and diverse cluster (MPD 1,355 SNPs) mainly of poultry origin, although three human isolates were included in the same cluster (Cluster 1, Fig. S2b).

The second largest and most diverse cluster in terms of included ST groups (Cluster 6; n = 21 isolates, n = 4 STs), primarily included ST10 Cplx isolates (n = 15) with ST744 as the most prevalent (n = 8 isolates) (Fig. 1). ST744-*bla*_SHV-12_ *E. coli* (n = 8/100) isolated from breeders, broilers and carcasses of chain C formed two clusters with an MPD of respectively six and 21 SNPs (Fig. S1c). The ESBL/pAmpC isolates of ST744 at Enterobase (n = 79) were mainly of human origin, and none of the isolates carried *bla*_SHV-12_ or was of poultry origin. Our ST744 isolates clustered with a set of human isolates, which mainly carried *bla*_CTX-M-15_ (Cluster 4, Fig. S2c). We found further evidence for the distribution of ST744 in animals^18^ and humans^14,19^, which corroborates the omnipresence of ST10 Cplx members in both populations^19^.

All isolates of Cluster 2, including the novel ST9298 isolate and the closely related ST429 isolates (n = 7), harboured *bla*_CMY-2_ (Fig. 1). Four out of five isolates from one-day-old, imported Parent Stock (PS) chicks were assigned to ST429. The remaining ST429 isolates (n = 3), together with one isolate of the novel ST9298 isolated from a PS breeder, were subsequently recovered from broilers and a carcass and belonged to chains A and C (Fig. S1d). The MPD of SNPs (43–76 SNPs) among the isolates of each of the three identified clusters (Fig. S1d) were generally larger compared to the aforementioned ST groups, even for a set of three isolates that were isolated from the same PS breeder flock (Cluster 2, Fig. S1d). Hence, instead of direct clonal transmission, the repeated introduction and subsequent circulation of closely related ST429-*bla*_CMY-2_ isolates through the import of PS chicks in each broiler production cycle may be a more likely scenario. SNP analysis of ST429/ST9298 isolates of this study and Enterobase (n = 30) revealed that almost all isolates harboured *bla*_CMY-2,_ clustered together with an MPD of 76 SNPs and were predominantly of poultry origin (Cluster 1, Fig. S2d). The only exception in the ST429 dataset was one human *bla*_TEM-52_-carrying *E. coli*, which showed an MPD of 2,976 SNPs to the isolates of Cluster 1. The Enterobase isolates originated from various stages of the broiler production, including isolates from breeders and poultry meat (not displayed). This finding highlights the existence of a conserved ST429-*bla*_CMY-2_ lineage, which seems to have spread in the European poultry production.

### Resistance genes other than ESBL/pAmpC genes

Sulphonamide resistance genes were present in 89% (n = 89/100) of isolates with the *sul2* gene as most prevalent (Table 1). Aminoglycoside acetyltransferase genes (*aac(3)*-like), nucleotidyltransferase genes (*aadA*-like) and phosphotransferase genes (*strA*, *strB*, *aph(3′)*-like) were found in 87% of isolates. The tetracycline resistance genes *tet*(A) and *tet*(B) were found in 76% and 12% of the *E. coli*, respectively. Moreover, WGS data showed trimethoprim resistance conferred by *dfrA*-like genes in 45% of isolates, with *dfrA14* being the most prevalent. The chloramphenicol exporter gene *floR* was present in 21 out of the 43 isolates, which carried phenicol resistance genes, while *cmlA1*, *catA1* and *catB3* were also present. Plasmid-mediated quinolone resistance (PMQR) genes were identified in 20% of the *E. coli*, specifically *qnrS1* (13%), *qnrS2* (4%) and *qnrB19* (2%). In addition, quinolone resistance was mediated by chromosomal mutations in quinolone resistance determining regions (QRDR) in 49% of isolates. The most prevalent mutations were those resulting in the amino acid exchanges Ser83Leu in GyrA (47%), followed by Ser80Ile in ParC (34%). Twelve isolates had combinations of PMQR genes and mutations in the *gyrA* and/or *parC* genes. In addition, 14 isolates harboured at least one of the macrolide resistance genes *mph*(A) or *mph*(B), which were found in twelve and two isolates, respectively. Furthermore, the rifampicin resistance gene *arr-3*, and the *aac(6′)Ib-cr* gene, which confers resistance to both fluoroquinolones and aminoglycosides, were found in all four ST2179 isolates of this study (Table 1). Although we did not confirm resistance phenotypes by susceptibility testing, previous studies showed high accordance between resistance phenotype and genotype^20,21^. Multi-drug resistance, which is frequently observed in ESBL/pAmpC-EC^22,23^, is crucial for their selection and dissemination by the use of non-β-lactam antimicrobial agents and thus may help to explain the high prevalence of ESBL/pAmpC-EC in broilers even in the absence of cephalosporin use^24^. Indeed in our case, all PS and fattening broiler flocks, except for three, received standard antimicrobial treatments for therapeutic reasons, which mainly included the administration of amoxicillin for ~ 5 days although occasional use of enrofloxacin and oxytetracycline was recorded as well^13^. Therefore, antimicrobial administration may have led to the selection of resistant isolates in the chicken gut and enabled their further dissemination^24^. However, alternative explanations may exist since ESBL/pAmpC genes can be maintained in bacterial populations even in the absence of antimicrobial selection pressure through conjugation and as a result of their low fitness cost^25^. Nevertheless, antimicrobial administration seems to play a central role in the complex mechanisms that modulate the abundance of ESBL/pAmpC plasmids in a population through the modulation of pathways that lead to their positive or negative frequency-dependent selection^25^.

### Virulence genes

**Figure 2:**
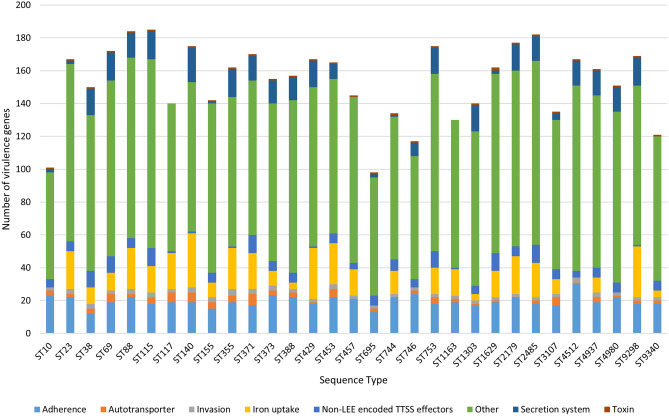
Distribution of virulence genes by function in the sequence types identified in this study.

Overall, 320 different virulence genes (VGs) were identified in the 100 sequenced *E. coli* and individual isolates carried a median of 170 VGs. A total of 56 virulence genes were ubiquitous. These included the curli fibers genes (*csg*), common-pilus genes (*ecp*), enterobactins (*ent*), ferrienterobactins (*fep*), the fimbrial proteins *fimD* and *fimG*, flagellar proteins of the families *flg* and *fli* and the flagellar motor proteins *motAB*. Only a few of the VGs showed an association with specific STs. While screening for a total of 89 adherence related VGs, we detected an average of 20 adherence-related VGs per isolate (Fig. 2). The adherence-conferring *pix* pili genes (*pixCDFXH*) were found in 13/100 isolates and are in general connected to uropathogenic *E. coli* (UPEC)^26^. Moreover, the *etp* gene cluster (which codes for a type II secretion system) and the s*tcE* protease gene, mostly associated with enterohaemorrhagic *E. coli* O157:H7^27,28^, were found in two and one isolates, respectively (File S1). Autotransporters showed a heterogeneous distribution among the various STs. From this category, the *upaG* adhesion gene, associated with UPEC, was found in 41 isolates^29^. Additionally, the temperature-sensitive hemagglutinin autotransporter *tsh* gene was identified in ST371 isolates (n = 2/100) and has previously been linked to avian pathogenic *E. coli* (APEC)^30^. With regard to the 33 iron uptake VGs analysed, the *sit-*like iron transporter and *iuc*-like aerobactin genes were associated with 83.8% (n = 26/31) and 61.3% (n = 19/31) of the STs identified in this study, respectively. Furthermore, the toxin haemolysin E (*hlyE*) gene was present in the majority of STs (n = 29/31) and in 83/100 isolates. Most isolates showed the presence of VGs with miscellaneous functions (Fig. 2), such as the increased serum survival (*iss*) gene (n = 87/100), which is a crucial VG of extra-intestinal pathogenic *E. coli* (ExPEC)^31^, as well as *hma* (n = 9/100) that encodes a haem acquisition protein and plays a critical role in the colonisation of the urinary tract^32^. Additionally, the ESBL/pAmpC-EC were assigned to pathotypes based on previously defined criteria (File S1)^33–36^. Interestingly, 56 isolates belonging to 18 different STs were classified as atypical enteroaggregative *E. coli* (aEAEC) as they possessed *aatA* but lacked the *aggR* regulon^35,37^. About one third (34%) of the aEAEC group was represented by ST457 isolates (n = 19/25). In the study of Bamidele et al.^36^, eight out of 31 adults suffering from travellers’ diarrhoea carried aEAEC that harboured only *aatA* and not *aggR* or *aaiC* and were positive in the Hep-2 cell adherence test, which remains the gold-standard in the assessment of aEAEC/EAEC pathogenicity^36,37^. Both typical and atypical EAEC have been predominantly associated with paediatric diarrhoea in developing countries^35,37^. Further, 51 isolates from 12 STs were characterised as ExPEC [presence of ≥ 2 of *pap* (P fimbriae), *sfa/foc* (S/F1C fimbriae), *afa*/*dra* (Dr binding adhesins), *iutA* (aerobactin receptor), and *kpsMT II* (group 2 capsule synthesis)]^31,33^. All ST457 isolates (n = 25/25) made up half (49%) of the ExPEC group. Moreover, 39 isolates, belonging to 13 different STs, were classified as avian pathogenic *E. coli* (APEC) [presence of *iutA*, *hlyF* (haemolysin F), *iss*, *iroN* (siderophore receptor), *ompT* (outer membrane protein T)]^34^. The APEC group mainly (38.5%) consisted of ST744 (n = 8/8) and ST429/ST9298 (n = 7/8) isolates. Interestingly, 42 isolates showed hybrid pathotypes, with 30 isolates from seven STs displaying the aEAEC/ExPEC pathotype (File S1). This latter subgroup was predominantly represented by the isolates of ST457 (n = 19/25), suggesting an association of this ST with the aEAEC/ExPEC pathotypes, followed by ST155 (n = 4/10). While the possession of ExPEC VGs was expected for the APEC and ExPEC pathotypes, it was less anticipated for aEAEC, since it is a potential intestinal pathogenic *E. coli* (InPEC) pathotype. A recent study suggested that such hybrid pathotypes are dominant in human faecal microbiota and are usually undetected by routine diagnostic tests^38^. Although prediction of pathogenicity is fundamental for QMRA models, the distinction between commensals and pathogens has become a bigger conundrum in the WGS era. For example, disease-associated VGs were identified in probiotic bacteria with a long history of safe use^39^. Another study suggested that pathogenicity may be linked to genome reduction rather than the acquisition of certain VGs^40^. It is therefore likely that traditional pathotyping schemes will be modified in the future and that multi-omic pathogenicity predictive models that consider the presence and expression of all genes rather than small sets will gain more ground as genome databases grow^11^. Nevertheless, the virulence gene repertoire of ESBL/pAmpC-EC reported here and in other studies, may explain their adaptation to and persistence in various niches, such as the poultry environment^41^.

### Genomic localisation of ESBL/pAmpC genes

ESBL/pAmpC genes were often associated with plasmids belonging to specific incompatibility groups and in some cases associations with certain STs were also observed. The most dominant ESBL/pAmpC gene/plasmid association was the presence of *bla*_CMY-2_ on IncK2 plasmids, which was found in 27% of the sequenced isolates, belonging to a variety of different STs (11 out of 31 STs) although predominantly in ST429 (7 out of the 27 *bla*_CMY-2_-carrying IncK2 plasmids) (Table 1). Another striking association was the occurrence of *bla*_CTX-M-55_ on IncFIB/IncFII plasmids (25%), which was uniquely present in all isolates of ST457. The *bla*_CTX-M-1_ gene was mainly found on IncI1 plasmids (13%), but IncI2, IncFIB/IncFII and IncHI2 plasmids also harboured this gene (Table 1). Moreover, *bla*_SHV-12_ was found to be carried by IncX3 or IncI1 plasmids in 12% and 6% of the isolates, respectively (Table 1). For the four *bla*_CTX-M-65_-carrying ST2179 isolates, the gene was not associated with any plasmid replicons and we thus concluded its putative chromosomal localisation, which will be further investigated. The genetic context of the most dominant plasmid types is described below.

#### *IncK2 plasmids carrying bla*_*CMY-2*_

**Figure 3:**
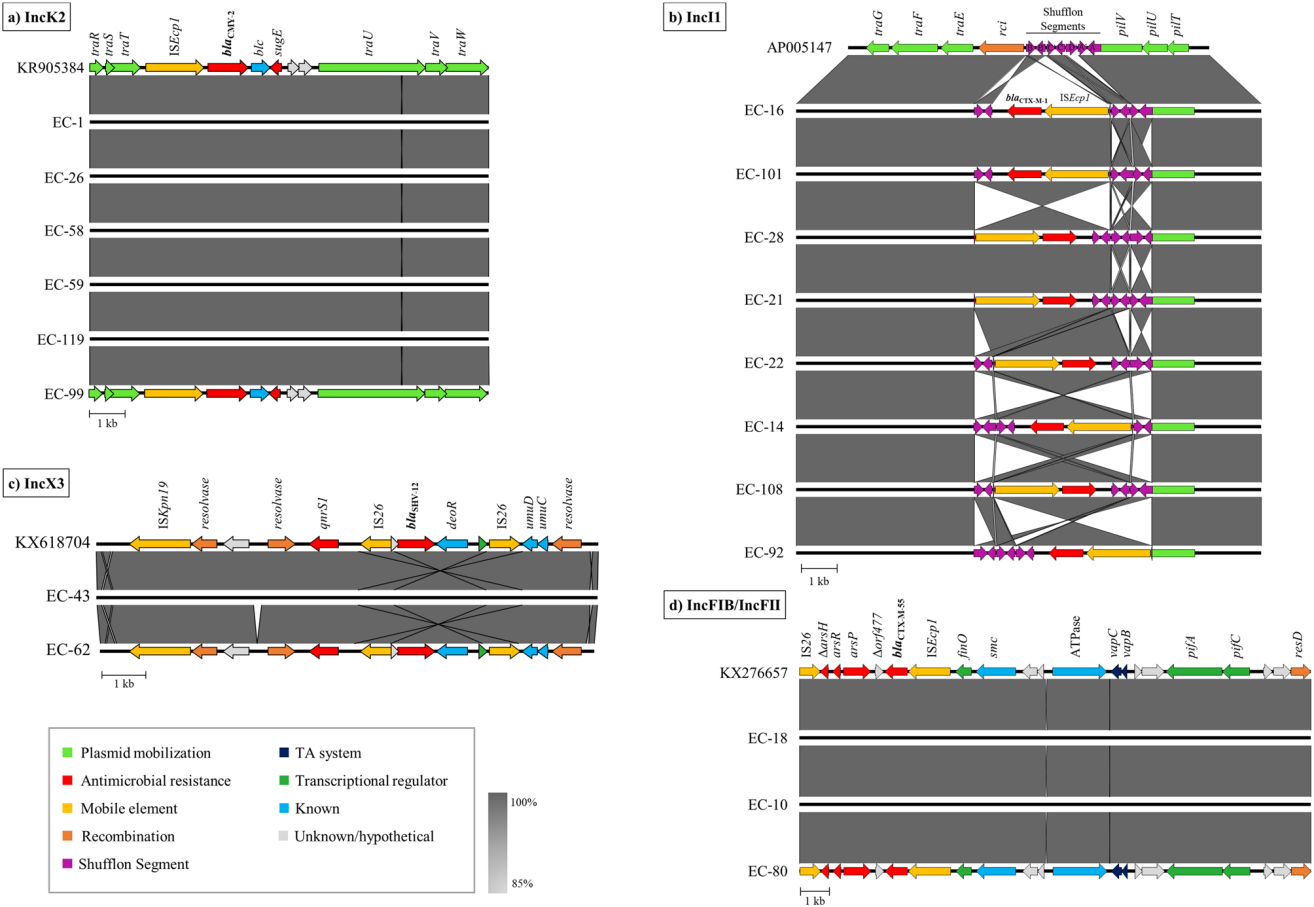
Linear comparison of (**a**) IncK2, (**b**) IncI1, (**c**) IncX3 and (**d**) IncFIB/IncFII plasmid regions carrying *bla*_CMY-2_, *bla*_CTX-M-1_, *bla*_SHV-12_ and *bla*_CTX-M-55_ genes, respectively, identified in our study. The open reading frames are represented with arrows, with the arrowhead indicating the direction of transcription. Their role in transfer, recombination, antimicrobial resistance, their association to mobile genetic elements and known or unknown functions are colour-coded. Areas shaded in grey indicate the percentage of nucleotide sequence identity.

In 27 of the thirty *E. coli* genomes harbouring *bla*_CMY-2_, the resistance gene was associated with the novel incompatibility group IncK2^42^. An approximately 12 kb large region including *bla*_CMY-2_ was highly conserved in all plasmids and was almost identical (99.81–100% nucleotide sequence identity) to that of plasmid pDV45 (GenBank accession no. KR905384.1). IS*Ecp1*-*bla*_CMY-2_-*blc*-*sugE* was integrated into the *tra* locus between the *traU* and *traT* genes in all cases (Fig. 3a). The two imperfect inverted repeats (IV-L: TGACGGTGATCCT; IV-R: AGCATCTCCGTCA) described by Seiffert et al.^42^ flanking this mobile element were also identified in those sequences. Recent studies reported the dissemination of the highly conserved cluster of IncK2 plasmids in diverse *E. coli* of various reservoirs^14,43^, although they seem to be dominant in broilers and broiler meat^44^. In our study, IncK2 plasmids were identified in isolates assigned to eleven different STs recovered from different stages of the production pyramid (Table 1, Fig. 3a). Interestingly, a further comparative analysis with ABACAS of all ST429-*bla*_CMY-2_ isolates of poultry origin present in Enterobase using plasmid pDV45 as reference, showed in all cases the presence of the aforementioned IS*Ecp1*-*bla*_CMY-2_-*blc*-*sugE* element integrated in IncK2 plasmids (data not shown). It is also noteworthy that plasmids with almost identical IncK2 backbones have been identified in *E. coli* isolated from hospitalised patients^42,44^. Furthermore, Donà et al.^45^ reported a similar IncK2 plasmid harbouring *mcr-1*, *bla*_TEM-1_ and *sul2* but not *bla*_CMY-2_ in *E. coli* from retail chicken meat in Switzerland. In our study, *bla*_CMY-2_ was the only resistance gene present on IncK2 plasmids with the exception of four plasmids that carried also *aac(3)-*VIa, *aadA1* and *sul1* (Table 1).

#### *IncI1 plasmids carrying bla*_*CTX-M-1*_

The *bla*_CTX-M-1_ gene was mainly detected on IncI1 plasmids (n = 13 of 17), which together with IncN plasmids, contribute to the spread of *bla*_CTX-M_ genes^46^. All *bla*_CTX-M-1_-carrying IncI1 plasmids belonged to plasmid MLST type pST3 or to closely related variants (Table 1). The genetic region containing *bla*_CTX-M-1_ (~ 12 kb) was highly conserved amongst all plasmids (99.96% nucleotide sequence identity) and the well-studied IncI1 plasmid R64 (GenBank accession no. AP005147) served as a reference (Fig. 3b). The mobile element IS*Ecp1-bla*_CTX-M-1_ was integrated in the shufflon region, between the *rci* shufflon-specific recombinase and the *pilV* gene, which represent a hotspot for DNA rearrangements^46^. Rci binds to specific recombination sites (*sfx* repeats) and causes the rearrangement of shufflon segments, which leads to variable arrangements of the shufflon region, even within the same plasmid preparation^47^, in what seems to be a non-random process^48^. It is, therefore, highly probable that the observed re-arrangements in the integration site of *bla*_CTX-M-1_ (Fig. 3b) do not represent multiple insertion events but rather recombination events due to Rci activity. The insertion of the module IS*Ecp1-bla*_CTX-M-1_ into the shufflon region seems to be common for IncI1 plasmids belonging to pST3, as shown in previous studies identifying this plasmid type from various sources^46,49–51^. A recent study reported identical *bla*_CTX-M-1_-carrying IncI1 pST3 plasmids from chicken, chicken meat, and bloodstream infections in humans^21^. Furthermore, additional resistance genes, mainly *tet*(A) and *sul2* (Table 1), were found on the IncI1-*bla*_CTX-M-1_ plasmids and their co-localisation with *bla*_CTX-M-1_ has been described before^21,45^.

#### *IncX3 plasmids carrying bla*_*SHV-12*_

The resistance gene *bla*_SHV-12_ was detected in 18 of the sequenced isolates and was carried by either IncX3 (n = 12/18) or IncI1 (n = 6/18) plasmids (Table 1). The genetic context of *bla*_SHV-12_ in IncX3 plasmids included two IS*26* elements in opposite orientation, with *qnrS1* being inserted upstream (Fig. 3c). The most similar sequences identified by BLASTn analysis of the ~ 11 kb region against the NCBI nucleotide database were ~ 48 kb large IncX3-*bla*_SHV-12_-*qnrS1* plasmids isolated from chicken faeces and a human patient in the Netherlands, reported by Liakopoulos et al. in 2018^52^ (e.g. GenBank accession no. KX618704, 99.82–100% nucleotide sequence identity). Similar IncX3 plasmids with the same *bla*_SHV-12_ surrounding region have been isolated from different countries and sources, including humans^52,53^. Despite of *bla*_SHV-12_ being mainly associated with IncI1 plasmids^54^, in our study we observed a higher occurrence of the IncX3 plasmid compared to IncI1 (Table 1). This shift has also recently been reported in the Netherlands, where a slow decrease of *bla*_SHV-12_-encoding IncI1 plasmids in favour of IncX3 plasmids was observed^52^. Except for *bla*_SHV-12_ and *qnrS1*, no additional resistance genes were co-located on IncX3 plasmids.

#### *IncFIB/FII plasmids carrying bla*_*CTX-M-55*_

Interestingly, *bla*_CTX-M-55_ was found only on IncFIB/FII plasmids of replicon sequence type F18:A-:B1 (n = 25/25). This variant of the CTX-M enzyme has been mainly reported in Asian countries, where it represents the second most common variant in *E. coli* isolated from companion and livestock animals^55,56^ and was only recently described in *E. coli* isolates of animal origin in France^18^.

BLASTn analysis of the region including *bla*_CTX-M-55_ yielded homologous (99–100% nucleotide sequence identity) *bla*_CTX-M-55_-carrying F18:A-:B1 plasmids isolated from clinical *E. coli* isolates of human origin in the United States (GenBank accession nos. CP041997.1, KX276657.1, CP029748.1) and wastewater in Canada (GenBank accession no. MK878525.1). The human related plasmids additionally carried *mcr* colistin resistance genes. Here we used the *mcr-1*-carrying plasmid pMR0516mcr isolated from a clinical ST457 isolate (GenBank accession no. KX276657.1)^17^ as a reference sequence (Fig. 3d). In all of our ST457 isolates, the *bla*_CTX-M-55_ gene is associated with IS*Ecp1*, within one of the most common genetic modules, IS*Ecp1*-*bla*_CTX-M-55_-*orf477*^57^, and is located between the *finO* gene, which is involved in the regulation of plasmid conjugation, and the arsenic resistance operon *ars* (Fig. 3d). Other co-located resistance genes (e.g*. bla*_TEM-1b_, *dfrA14*, *sul2*) were found in varying combinations in the majority of our IncFIB/FII plasmids (Table 1).

## Conclusions and outlook

Our WGS approach revealed a complex landscape of *E. coli* genotypes present in the broiler production. We demonstrated that 76% of the sequenced isolates shared four dominant plasmid types that harboured ESBL/pAmpC genes. The finding of diverse STs from various production stages, which share closely related plasmid backbones with identical ESBL/pAmpC gene insertions, is a strong indication that horizontal gene transfer via the exchange of plasmids that have been previously reported to be transmissible^42,46,52^, plays a fundamental role in the dissemination of ESBL/pAmpC genes across the broiler production pyramid^5^. In addition to β-lactamase genes, the sequenced isolates carried genes which conferred resistance to four other antimicrobial classes, on average, a fact that is relevant for public health and definitely needs to be considered in the hazard identification step of a QMRA. The co-localisation of several other resistance genes on ESBL/pAmpC-carrying plasmids might explain their persistence and propagation through selection pressure by non-3GC antimicrobial agents^49^. In addition to horizontal gene transfer, clonal expansion of several closely related ESBL/pAmpC-EC along the production pyramid was also observed and has been previously reported^58^. The identification of phylogenetically related isolates in distantly located farms, but also in subsequent production levels of the same production chain, further supports the argument of clonal transmission from the top of the pyramid to the lower levels of production. However, at least part of the ESBL/pAmpC-EC dissemination may be explained by indirect transmission events within the studied integrated production system (e.g*.* hatchery-to-farm or farm-to-farm transmission), which underlines the need to maintain and continuously monitor biosecurity protocols and thus prevent transmission of ESBL/pAmpC-EC by e.g*.* the movement of vehicles, equipment and personnel^5^. The importance of such measures is reflected by the fact that in accordance with other studies^22,59^, in our recent prevalence study^13^ we encountered the lowest occurrence of ESBL/pAmpC-EC in PS breeders, a production stage where increased biosecurity standards are implemented due to the higher value of these flocks compared to fattening broilers. Also, as Hiroi et al.^60^ showed, contamination of the environment of broilers with ESBL/pAmpC-EC may be a more important factor for their colonisation than the use of antimicrobial agents.

We previously discussed a potential introduction of *bla*_CMY-2_ isolates through the import of day-old PS chicks, based on the finding that all isolates from this production stage had the phylogroup B2-*bla*_CMY-2_ genotype^13^. Here, we show that at least a part of these isolates belong to ST429/ST9298, a highly conserved *bla*_CMY-2_-positive lineage, which seems to be prevalent in the European poultry production^14^. Results from the comparative analysis with ST429 genomes at Enterobase suggest the circulation of a conserved ST429/ST9298 lineage carrying *bla*_CMY-2_ on IncK2 plasmids in the poultry production of at least five European countries, which needs to be studied further in future investigations, considering also the pathogenic potential of this ST for poultry and its role in colibacillosis (n = 7/8 isolates were APEC). Additionally, these findings further support the notion that ESBL/pAmpC-EC may spread through the interconnected breeder stock supply chains^5,14,58^, which warrants their eradication in pedigree flocks and in hatcheries. This can be achieved by e.g*.* the complete cessation of ceftiofur administration in these flocks for prophylaxis^61^. Further, comparison with publicly available data proved that the occurrence of ESBL/pAmpC-EC in poultry is a dynamic phenomenon, since STs and ESBL/pAmpC genes not common in the European context, such as ST457 and *bla*_CTX-M-55_, can emerge and dominate in poultry. The fact that our *E. coli* isolates of ST457 and ST744 were closer related to human ones than to those originating from livestock (Fig. S2), raises questions about their ability to colonise the human gut upon exposure, transmit their resistance determinants, and/or exert their virulence potential. The latter aspect is especially relevant for ST457, which has a documented record of association with human disease and its isolates were assigned to defined pathotypes (ExPEC and/or EAEC) in our virulence analysis. Using an in vitro colon simulation system, Anjum et al.^62^ showed that *E. coli* isolated from human faeces and poultry meat were able to adapt and transmit their AMR plasmids to other *Enterobacteriaceae* in the human gut. Nevertheless, a recent study demonstrated that the dissemination of ESBL/pAmpC genes between animals and humans seems to be mediated by specific plasmid types rather than by expansion of successful *E. coli* clones^9^. This hypothesis is further supported by the detection of closely related plasmid backbones with identical ESBL/pAmpC gene insertion sites between the isolates of this study and those of human origin^17,21,42,52^.

Our isolate selection procedure was suitable for describing the diversity of ESBL/pAmpC-EC genotypes that may be encountered in the broiler production, studying the underlying transmission mechanisms and providing solid evidence of transmission events along the broiler production pyramid. However, our study has limitations. Quantitative estimates on transmission pathways as well as statistical associations between the identified genotypes, which are essential for QMRAs, cannot be inferred with our analysis since this would require the sequencing of a larger and more representative part of our initial collection of isolates^11^. Such approaches are hampered by the decreasing but still significant cost of WGS. The second limitation concerns the findings of our comparative genomic analysis, which should be interpreted with caution since potential bias in Enterobase data may exist. Our objective was not to make deductions on potential transmission events between the analysed hosts, which would also require thorough epidemiological data, but to gain insights from the host distribution and phylogenetic relatedness of dominant ESBL/pAmpC-EC lineages in the context of an extensive collection of publicly available genomes supported by basic metadata. The two major conclusions on the highly conserved, poultry-related ST429-IncK2-*bla*_CMY-2_ and the emerging ST457-*bla*_CTX-M-55_ were noteworthy outcomes of this analysis that ought to be investigated further.

Our results provide valuable insights into the characteristics of ESBL/pAmpC-producing *E. coli*, their plasmid contents and distribution in the broiler production chain. These results contribute to the growing amount of available high throughput data that will serve as the baseline for future food chain QMRA models. For example, the AMR profile, the genetic context of resistance genes and the virulence potential of isolates described in this study are relevant for guiding the risk identification and characterisation steps^11^. Given the key role of plasmids in the dissemination of ESBL/pAmpC genes elucidated here and in other studies^9^, exposure assessment modelling should definitely include, if not prioritise on, the transfer probability of ESBL/pAmpC plasmids, especially of those with a wide host-spectrum. The output of such thorough QMRAs will be the risk estimate of human exposure to resistance determinants via the (broiler) food chain and the identification of intervention measures with the maximum mitigation effect^11^.

## Methods

### Bacterial isolates

One-hundred isolates from a collection of ESBL/pAmpC-EC isolated from three production chains, namely A (n = 37), B (n = 32), and C (n = 31), of an integrated broiler company in Italy were included in this study^13^. Briefly, we sampled with cloacal swabs one PS flock per production chain at two time points; (i) one-day-old PS chicks upon arrival at the rearing farms and (ii) PS breeders at ~ 21 weeks of age during the laying period at the production farms. Afterwards, the offspring of the PS flock were sampled in four fattening farms per production chain at the start (one-day-old) and the end (~ 30-days-old) of the production cycle (broiler chicks and broilers, respectively). Carcasses from the sampled broilers were collected at the slaughterhouse after chilling and isolation of ESBL/pAmpC-EC was performed after rinsing the whole carcass with buffer peptone water^13^. *E. coli* phylogenetic groups and ESBL/pAmpC genes have been previously determined for these isolates by PCR and sequencing^13^. At least one isolate per production chain, production stage, ESBL/pAmpC gene, and phylogroup combination (e.g. isolate EC-1 in File S1, chain A/PS chicks/*bla*_CMY-2_/phylogroup B2) was randomly selected for sequencing to fully explore the diversity of ESBL/pAmpC-EC genotypes across the production pyramid and identify potential transmission events. The 100 selected isolates originated from the following production stages: PS chicks (n = 5), PS breeders (n = 8), broiler chicks (n = 26), broilers (n = 28) and slaughterhouse/carcass (n = 33) (File S1).

### Whole genome sequencing and in silico typing

DNA extraction and sequencing were carried out at two different sites. At the first site, DNA was isolated from 32 isolates with the Master Pure Genomic DNA-Purification Kit (Epicenter, USA), libraries were prepared with the Nextera XT library preparation kit (Illumina, USA) and sequencing on an Illumina MiSeq platform using the MiSeq v3 reagent kit (Illumina) with 2 × 300 bp paired-end reads. At the second site, DNA was isolated from 68 isolates with the Invisorb Spin Tissue Mini Kit (Invitek, Germany), library preparation was done with the Nextera XT library preparation kit and sequencing on an Illumina HiSeqX platform with 2 × 150 bp paired-end reads at a private company (Macrogen, Korea).

Raw reads were directly submitted for processing at the Enterobase database^63^. The Enterobase backend pipeline assures high quality assemblies by including reads pre-processing and post-correction of SPAdes^64^ assemblies (https://enterobase.readthedocs.io/). Assemblies that did not meet the quality control criteria were not used for downstream analysis. Assembly statistics (coverage, N50, genome size and contig number) and accession numbers (Enterobase barcodes) for each strain can be found in File S1. Further, sequenced isolates were genotyped in silico with regard to acquired resistance genes and chromosomal mutations (ResFinder 3.2 and PointFinder 3.1, respectively)^20^, as well as plasmid replicon types (PlasmidFinder 2.0)^65^ and pSTs when available (pMLST 2.0)^65^, using the tools at default settings, whereas *E. coli* STs were extracted from the Enterobase automated pipeline, which follows the Achtman MLST scheme.^66^ Presence of VGs was assessed with BLAST^67^ alignments (E < 10^–5^) to the Virulence Factors Database (VFDB)^68^ extended with VGs from literature (*hlyF*, *iss*, *ompT*)^31,34^. Based on their virulence gene content, isolates were assigned to known pathotypes^33–36^. A VG was considered to be associated to a given ST if at least 80% of the isolates included in that ST carried the specific VG.

### Plasmid characterisation

In several isolates, the ESBL/pAmpC gene-carrying plasmid was identified by the presence of the resistance gene and the plasmid replicon on the same contig. Otherwise, the contigs containing ESBL/pAmpC genes but missing the plasmid replicon were compared by BLASTn^67^ (default settings) to the most similar, complete reference plasmids available in GenBank database. To identify all the contigs corresponding to particular plasmids, the draft genome of each isolate and the reference plasmid were aligned with ABACAS^69^ as previously described^70^. Identification of the genetic context of ESBL/pAmpC genes was done by aligning the identified plasmid contigs against the reference plasmid in Geneious 10.1.3, using the multiple alignment tool and default settings. EasyFig^71^ was used to visualise aligned plasmid regions of interest.

To confirm results obtained in silico, plasmid transformation experiments were performed for at least one isolate with a specific ST/ESBL/pAmpC gene combination, resulting in a total of 32 plasmid transformations. Briefly, plasmid DNA was extracted with the alkaline lysis method^72^, electro-competent DH5α *E. coli* cells (Invitrogen, Denmark) were prepared according to Smith et al*.*^73^ and transformed by electroporation utilizing an Eppendorf Eporator (Eppendorf, Germany). Potential transformants harbouring an ESBL/pAmpC-carrying plasmid were isolated on LB agar (Microbiol, Italy) containing 1 mg/L cefotaxime. The incompatibility group of plasmids transferred from the wild type isolates was determined by PCR-based replicon typing (PBRT)^74,75^.

### Phylogenetic analysis

The phylogeny of the sequenced isolates was deduced by a SNP-based mapping analysis. A suitable reference was selected among all complete *E. coli* genomes (n = 983) (https://www.ncbi.nlm.nih.gov/genome/167) using Mash^76^ to find the reference genome with the least Mash distance from (i) all sequenced isolates and (ii) all isolates of each dominant ST group. The phylogenetic analysis was extended with all publicly available genomes at Enterobase, belonging to the most dominant STs of our collection and with a confirmed presence of ESBL/pAmpC genes by local ResFinder analysis. *E. coli* genomes were mapped to the selected reference (File S2) and a maximum likelihood SNP tree was created with CSI phylogeny^77^ using default settings. Cluster calculation was performed with the hierBAPS module of the Bayesian Analysis of Population Structure (BAPS) software v6.0^78^. BAPS clusters were assigned based on the output SNP alignment of CSI phylogeny with 2 levels of hierarchy and a maximum number of cluster (K) equal to the number of ST groups (n = 31) in the phylogenetic analysis of sequenced isolates, and equal to the number of isolates in the phylogenetic analysis of each ST group. All phylogenetic trees (Figs. 1, S1 and S2) generated with CSI phylogeny were visualised as phylograms and annotated with metadata in CLC Genomics Workbench version 12.0 (Qiagen, Denmark). The Mash-based ranking of complete reference genomes and their NCBI accession numbers, the barcodes of Enterobase genomes used for the comparative genomic analysis and the statistics of the SNP analysis (e.g*.* % reference coverage, number of SNPs) can be found in File S2.

### Ethics statement

Collection of cloacal swabs from live birds in the study of Apostolakos et al.^13^, was done by veterinarians of the private poultry company as part of the routine monitoring of the flock health status and conducted in compliance with good veterinary practices. This routine monitoring does not require approval by an institutional and/or licensing committee.

**Table 1:** Characteristics of 100 ESBL/pAmpC-EC isolates subjected to whole genome sequencing.

ESBL/ pAmpC gene	Plasmid Replicon type [no. of isolates]	pMLST/RST	MLST [no. of isolates]	Additional resistance genes located on ESBL/pAmpC gene-carrying plasmids^c^	Other resistance genes^d^
*bla*_CMY-2_	IncB/O/K/Z [27]^a^	–	ST429 [7]	*aac(3)-*VIa*, aadA1*, sul1*	*tet(A)*
			ST9298 [1]	–	*aac(3)-*VIa, *aadA1*, *tet(A)*, *sul1*
			ST155 [5]	–	*bla*_TEM-1b_*, *aac(3)-*VIa, *aadA1*, *tet(A)*, *sul1*
			ST10 [3]	–	*bla*_TEM-1b_*, *aac(3)-*VIa*, *aadA1*, dfrA1*, tet(A)*, sul1*
			ST140 [2]	*aac(3)-*VIa*, aadA1*, sul1*	*tet(A)*
			ST2485 [2]		*aac(3)-*VIa, *aadA1*, *tet(A)*, *sul1*
			ST371 [2]	*aac(3)-*VIa, *aadA1*, *sul1*	*tet(A)*
			ST38 [2]	–	*aac(3)-*VIa*, aadA1*, aph(3′)-Ic*, *strA*, *strB*, *tet(B)*, sul1*, *sul2*
			ST1163 [1]	–	*aac(3)-*VIa, *aadA1*, *sul1*
			ST373 [1]	–	–
			ST9340 [1]	–	*bla*_TEM-1b_, *aac(3)-*VIa, *aadA1*, *tet(A)*, *sul1*, *dfrA1*
	IncA/C_2_ [3]	pST3	ST355 [2]	–	*aac(3)-*VIa, *aadA1*, *strA*, *strB*, *floR*, *tet(A)*, *sul1*, *sul2*
			ST88 [1]	–	*bla*_TEM-1b_, *aac(3)-*VIa, *aadA1*, *aadA2*, *strA*, *strB*, *cmlA1*, *floR*, *tet(A)*, *sul1*, *sul2*, *sul3*
*bla*_CTX-M-55_	IncFIB/IncFII [25]	F18:A-:B1	ST457 [25]	*bla*_TEM-1b_*, aph(3′)-Ia*, tet(A)*, sul2*, *dfrA1*4*	aac(3)-IIa*, aadA1*, strA*, strB*, floR*, sul3*
*bla*_CTX-M-1_	IncI1 [13]	pST3^b^	ST115 [3]	aadA5*, tet(A)*, sul2*, *dfrA1*7*	–
			ST117 [3]	*tet(A)*, *sul2*	–
			ST155 [2]	*tet(A)*, *sul2*	*bla*_TEM-1b_, *aadA5*, *aph(3′)-Ia*, *strA*, *strB*, *catA1*, *tet(B)*, , *dfrA1*7
			ST453 [2]	*tet(A)*, *sul2*	*bla*_TEM-1b_, *strA*, *strB*, *dfrA1*4
			ST23 [1]	*tet(A)*, *sul2*	*bla*_TEM-1b_, *aadA1*, *sul3*
			ST2485 [1]	*tet(A)*, *sul2*	*bla*_TEM-1b_
			ST753 [1]	*tet(A)*, *sul2*	*bla*_TEM-1b_, *qnrS1*
	IncI2 [2]	–	ST38 [2]	–	*aadA1*, *strA*, *strB*, *tet(A)*, *mph*(B), *sul1*, *sul2*, *dfrA1*
	IncFIB/IncFII [1]	Unknown	ST4937 [1]	–	*aadA1*, *strA*, *strB*, *catA1*, *tet(A)*, *mph*(A), *sul1*, *dfrA1*
	IncHI2 [1]	Unknown	ST3107 [1]	–	aac(3)-IId, *tet(A)*, *sul2*, *dfrA1*4, *dfrA1*7
*bla*_SHV-12_	IncX3 [12]	-	ST744 [8]	*qnrS1*	*bla*_TEM-1b_, *aadA5*, aph(3′)-Ia*, *strA*, *strB*, *catA1*, *tet(B)*, *mph*(A), *sul1*, *sul2*, *dfrA1*7
			ST1629 [3]	*qnrS1*	*tet(A)*, *sul1*
			ST746 [1]	*qnrS1*	*cml*, *qnrA1*, *sul1*, *dfrA5*
	IncI1 [6]	pST26	ST155 [3]	–	*aadA1*, *aadA2*, *cmlA1*, *tet(A)*, *sul3*
		pST26	ST4512 [1]	–	*aadA1*, *aadA2*, *cmlA1*, *tet(A)*, *sul3*
		pST95	ST117 [1]	–	*aadA1*, *aadA2*, *cmlA1*, *tet(A)*, *sul3*
		pST3	ST1303 [1]	–	*bla*_TEM-1b_, *tet(A)*, *qnrB19*
*bla*_CTX-M-65_	Chromosome [4]	–	ST2179 [4]	–	*bla*_OXA-1_, *bla*_TEM-1a_*, *bla*_TEM-1b_*, *aadA1*, *aadA2*, aadA5*, strA*, strB*, *catB3*, *cmlA1*, tet(A)*, *qnrS2*, *mph*(A)*, sul1*, sul2*, *sul3*, *dfrA1*7*, arr-3*, *aac(6′)Ib-c*
*bla*_TEM-52b_	IncX1 [2]	–	ST388 [1]	–	*qnrB19*
			ST695 [1]	–	–
*bla*_CTX-M-2_	IncQ1 [2]	–	ST4980 [2]	–	*bla*_TEM-1b_, *aadA1*, *strA*, *strB*, *tet(A)*, *sul1*, *sul2*, *dfrA1*
*bla*_CTX-M-15_	IncY [1]	–	ST69 [1]	–	*bla*_TEM-1b_, *strA*, *strB*, *tet(A)*, *qnrS1*, *sul2*
*bla*_SHV-2_	Untypable [1]	–	ST69 [1]	–	*aadA1*, *aadA2*, *cmlA1*, *tet(A)*, *sul3*

^a^The IncB/O and IncK replicons were identified by PBRT. Further analysis indicated that these plasmids belong to the novel incompatibility group IncK2^42^.

^b^Four isolates had truncated *ardA* genes in their assemblies but the other four alleles (*pilL*2, *repI*12, *sogS*1 and *trbA*4) were those of pST3. One isolate had also a truncated *ardA* gene and a novel *sog*S allele, differing from *sog*S1 by the mutation C24T. One isolate represented a novel IncI1 pST, closely related to pST3 (${ardA}1\rightarrow{ardA}7$).

^c^Resistance genes in this column were found on the ESBL/pAmpC gene-carrying plasmids after analysis with ABACAS. The following plasmid types, found on 76% of isolates, were studied: *bla*_CMY-2_-IncB/O/K/Z, *bla*_CTX-M-55_-IncFIB/IncFII, *bla*_CTX-M-1_- IncI1, *bla*_SHV-12_-IncX3. Resistance genes marked with an asterisk indicate that they were not present in all the corresponding plasmids.

^d^Resistance genes marked with an asterisk indicate that they were not present in all the isolates of the corresponding ST.

## Supplementary information

Supplementary information

Supplementary file S1

Supplementary file S2

## Data Availability

All data generated or analysed during this study are included in this published article and its Supplementary Information files.
