# Supplementary material for: High-resolution characterisation of ESBL/pAmpC-producing Escherichia coli isolated from the broiler production pyramid
Source: Sci Rep. 2020 Jul 7;10:11123. doi: 10.1038/s41598-020-68036-9 (PMC7341882; doi:10.1038/s41598-020-68036-9)
Supplement: Supplementary file 1 — Supplementary information [file 41598_2020_68036_MOESM1_ESM.pdf]

High-resolution characterisation of ESBL/pAmpC-producing *Escherichia coli* isolated from the broiler production pyramid

Ilias Apostolakos[1], Claudia Feudi[2], Inga Eichhorn[2], Nicola Palmieri[3], Luca Fasolato[1], Stefan Schwarz[2], Alessandra Piccirillo[1]*

[1]Department of Comparative Biomedicine and Food Science, University of Padua, Padua, 35020, Italy.

[2]Institute of Microbiology and Epizootics, Centre for Infection Medicine, Department of Veterinary Medicine, Freie Universität Berlin, Berlin, 14163, Germany.

[3]University Clinic for Poultry and Fish Medicine, Department for Farm Animals and Veterinary Public Health, University of Veterinary Medicine, Vienna, 1210, Austria.

[*]alessandra.piccirillo@unipd.it

a) ST457

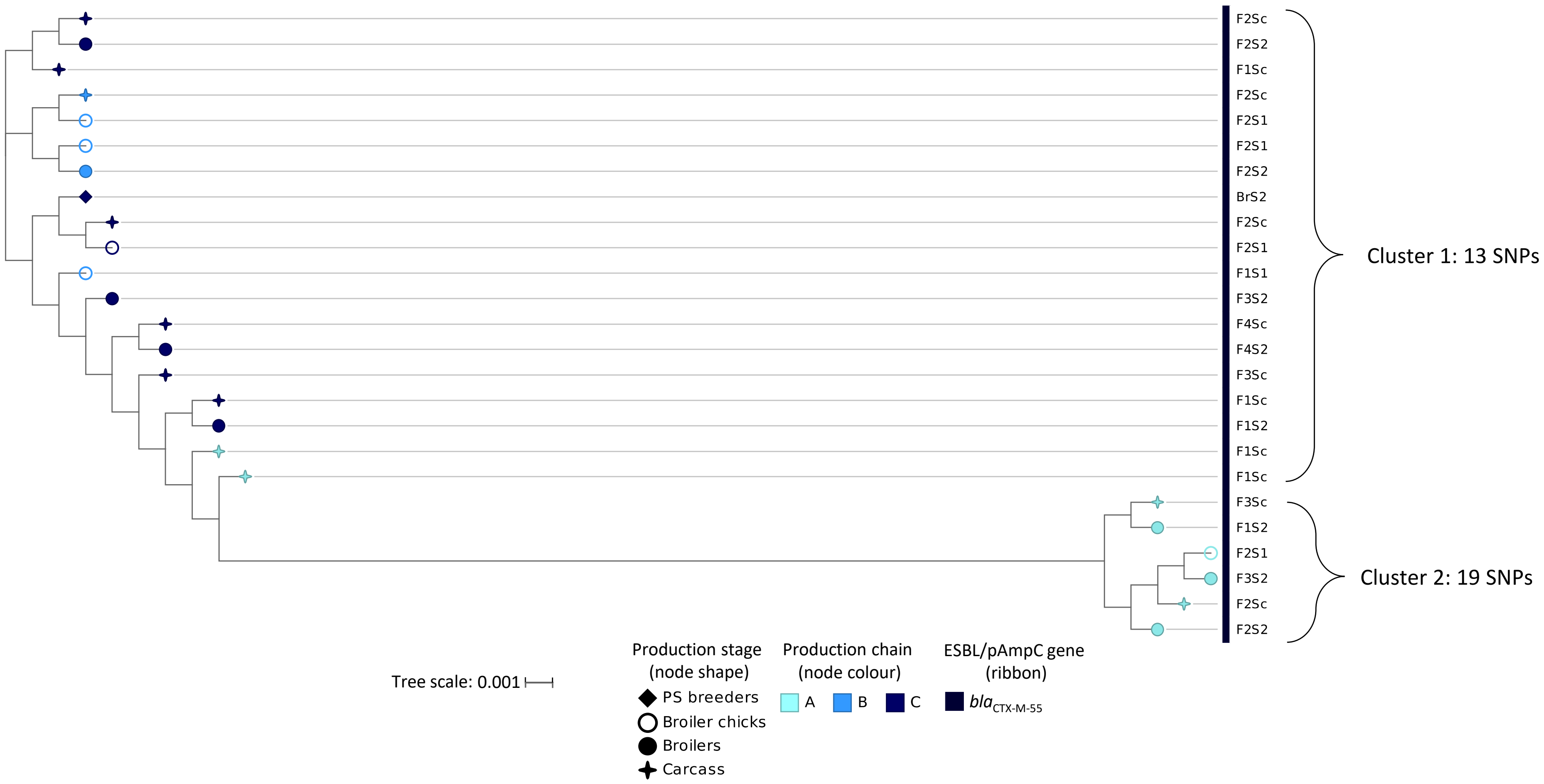
F2Sc
F2S2
F1Sc
F2Sc
F2S1
F2S1
F2S2
BrS2
F2Sc
F2S1
F1S1
F3S2
F4Sc
F4S2
F3Sc
F1Sc
F1S2
F1Sc
F1Sc
F3Sc
F1S2
F2S1
F3S2
F2Sc
F2S2
Cluster 1: 13 SNPs
Cluster 2: 19 SNPs
Tree scale: 0.001
Production stage (node shape)
PS breeders
Broiler chicks
Broilers
Carcass
Production chain (node colour)
A
B
C
ESBL/pAmpC gene (ribbon)
$bla_{CTX-M-55}$

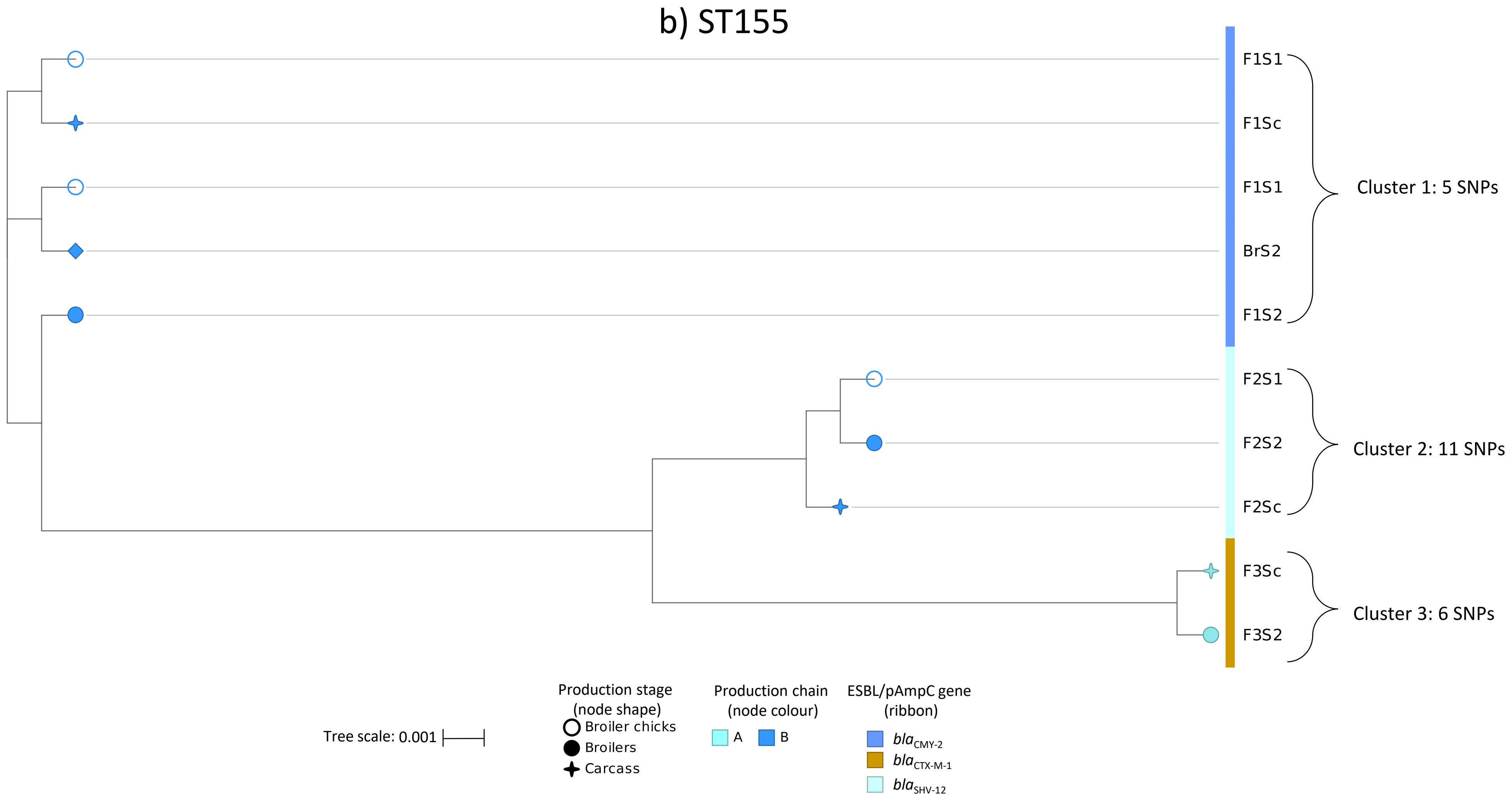

b) ST155
F1S1
F1Sc
F1S1
BrS2
F1S2
F2S1
F2S2
F2Sc
F3Sc
F3S2
Cluster 1: 5 SNPs
Cluster 2: 11 SNPs
Cluster 3: 6 SNPs
Tree scale: 0.001
Production stage (node shape)
Broiler chicks
Broilers
Carcass
Production chain (node colour)
A
B
ESBL/pAmpC gene (ribbon)
bla_{CMY-2}
$bla_{CTX-M-1}$
bla_{SHV-12}

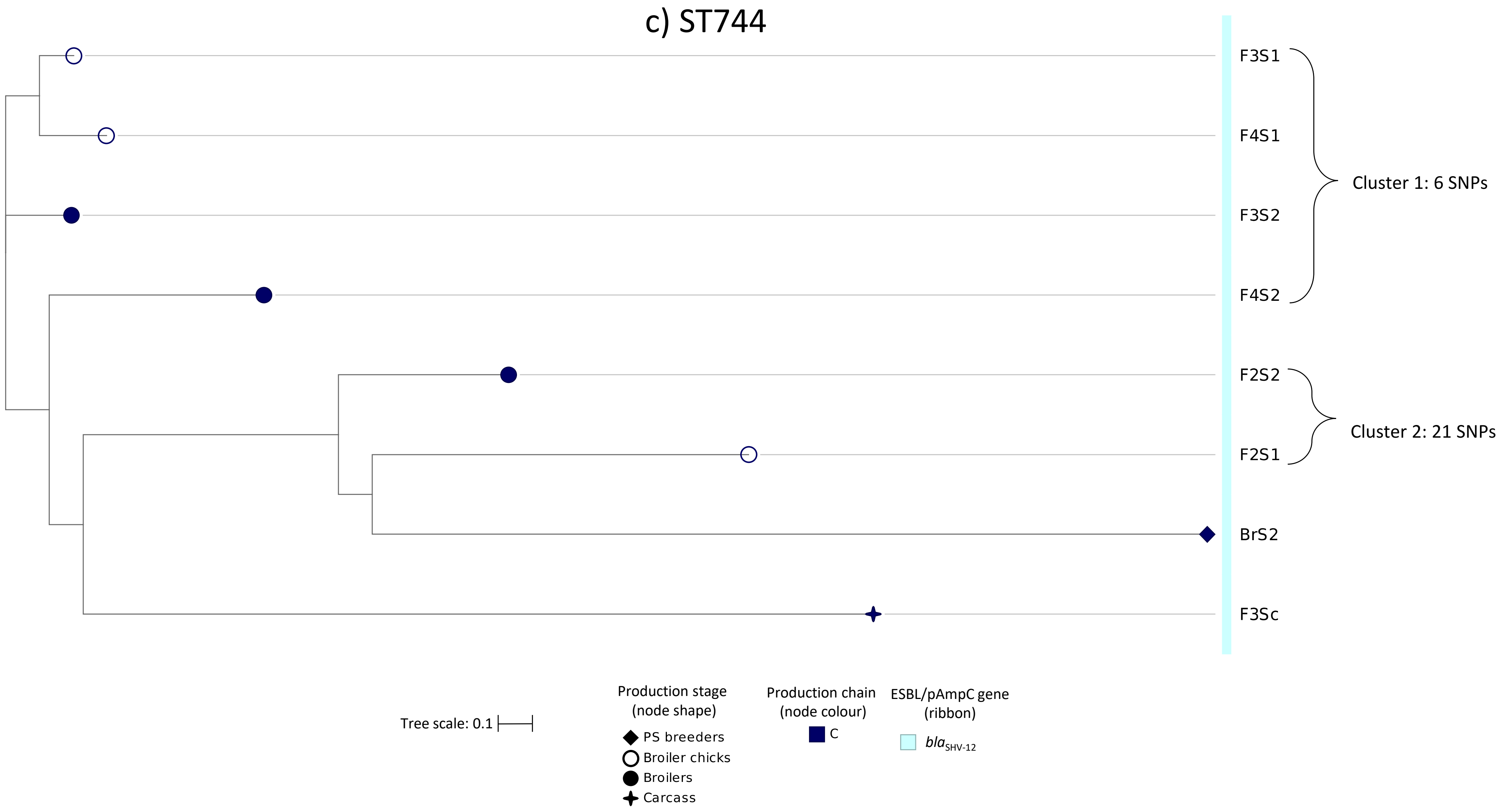
c) ST744
F3S1
F4S1
F3S2
F4S2
F2S2
F2S1
BrS2
F3Sc
Cluster 1: 6 SNPs
Cluster 2: 21 SNPs
Tree scale: 0.1
Production stage (node shape)
PS breeders
Broiler chicks
Broilers
Carcass
Production chain (node colour)
C
ESBL/pAmpC gene (ribbon)
bla_{SHV-12}

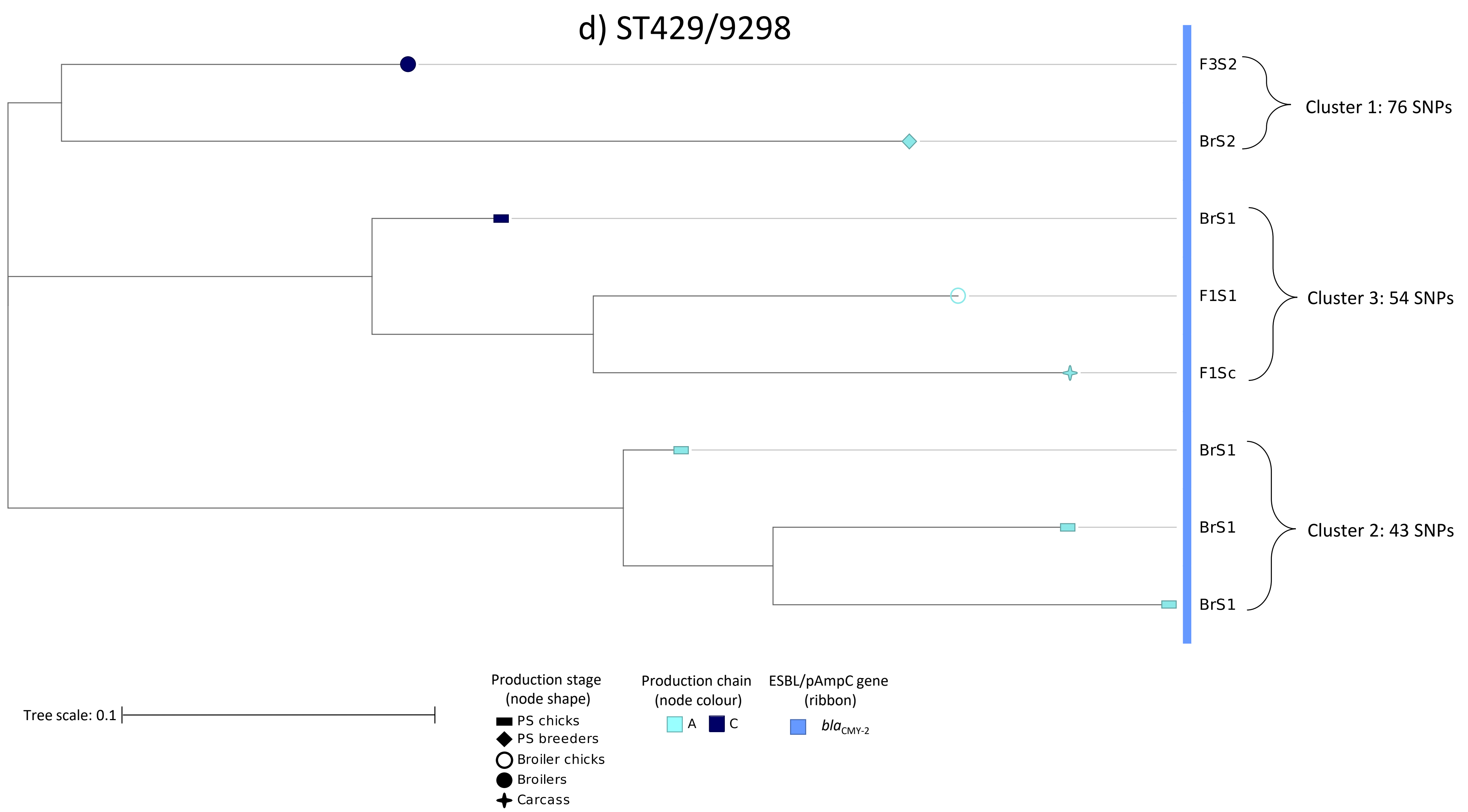

d) ST429/9298
F3S2
BrS2
BrS1
F1S1
F1Sc
BrS1
BrS1
BrS1
Cluster 1: 76 SNPs
Cluster 3: 54 SNPs
Cluster 2: 43 SNPs
Tree scale: 0.1
Production stage (node shape)
PS chicks
PS breeders
Broiler chicks
Broilers
Carcass
Production chain (node colour)
A
C
ESBL/pAmpC gene (ribbon)
blaCMY-2

Supplementary Figure S1. Phylogenetic analysis of the most prevalent sequence types (STs) of this study a) ST457, b) ST155, c) ST744, d) ST429/ST9298. SNP analysis was done with CSI phylogeny. Colour-coded nodes and ribbons respectively represent the production chains and ESBL/pAmpC genes of isolates. SNPs numbers indicate the median of pairwise SNP differences between the isolates of the corresponding cluster marked with braces. Text next to nodes denotes farms and stages at which isolates were obtained, with the following coding system: BrS1, PS chicks; BrS2, PS breeders; F1-4S1, farm 1-4 broiler chicks; F1-4S2, farm 1-4 broilers; F1-4Sc, farm 1-4 carcasses. Scale bar refers to the branch lengths, which are measured in the number of substitutions per site. Tree nodes are ordered after their increasing branch lengths.

a) ST457

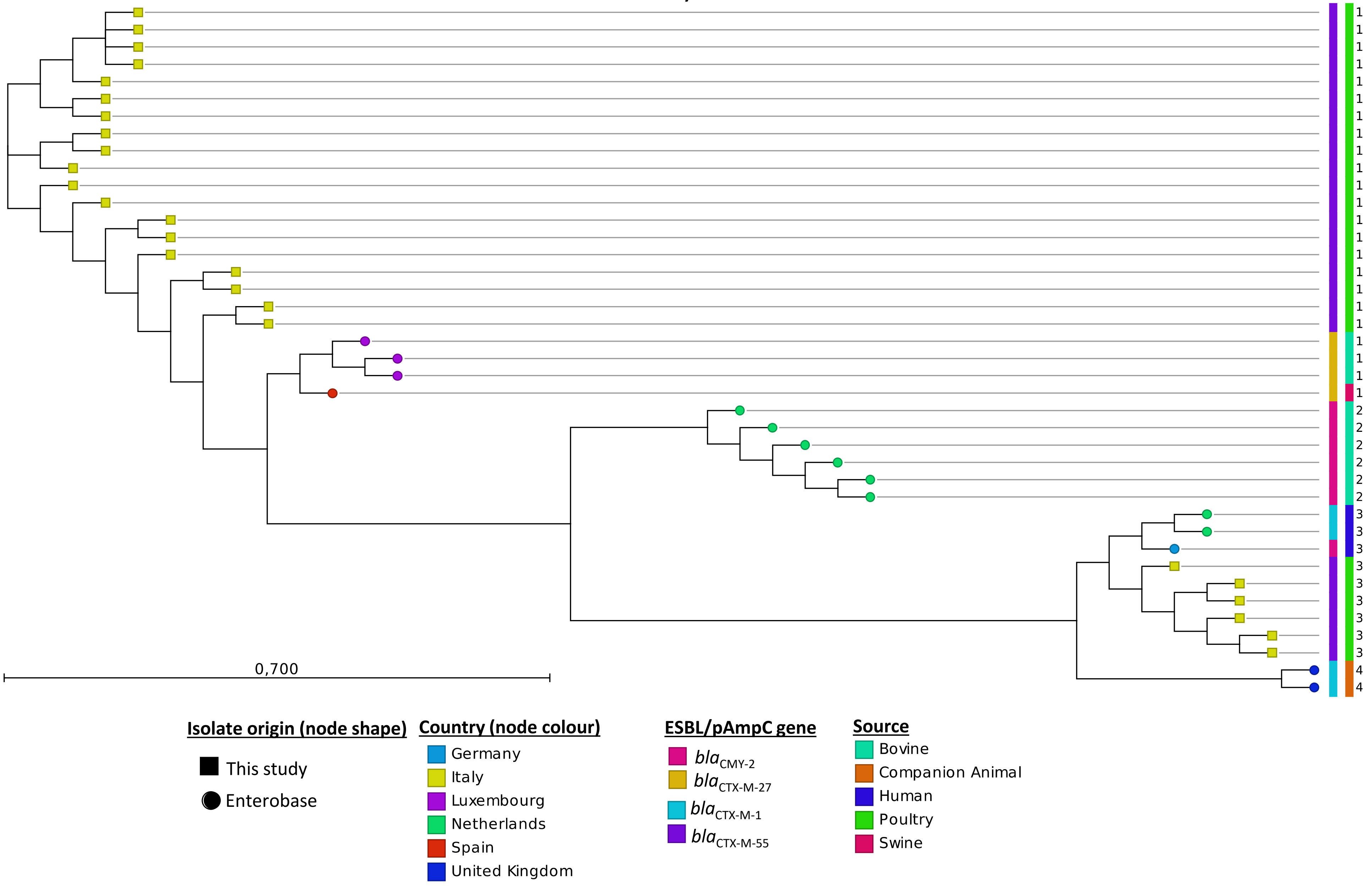

ESBL/pAmpC gene
Source
Cluster ID
0,700
Isolate origin (node shape)
This study
Enterobase
Country (node colour)
Germany
Italy
Luxembourg
Netherlands
Spain
United Kingdom
ESBL/pAmpC gene
bla_{CMY-2}
$bla_{CTX-M-27}$
$bla_{CTX-M-1}$
$bla_{CTX-M-55}$
Source
Bovine
Companion Animal
Human
Poultry
Swine

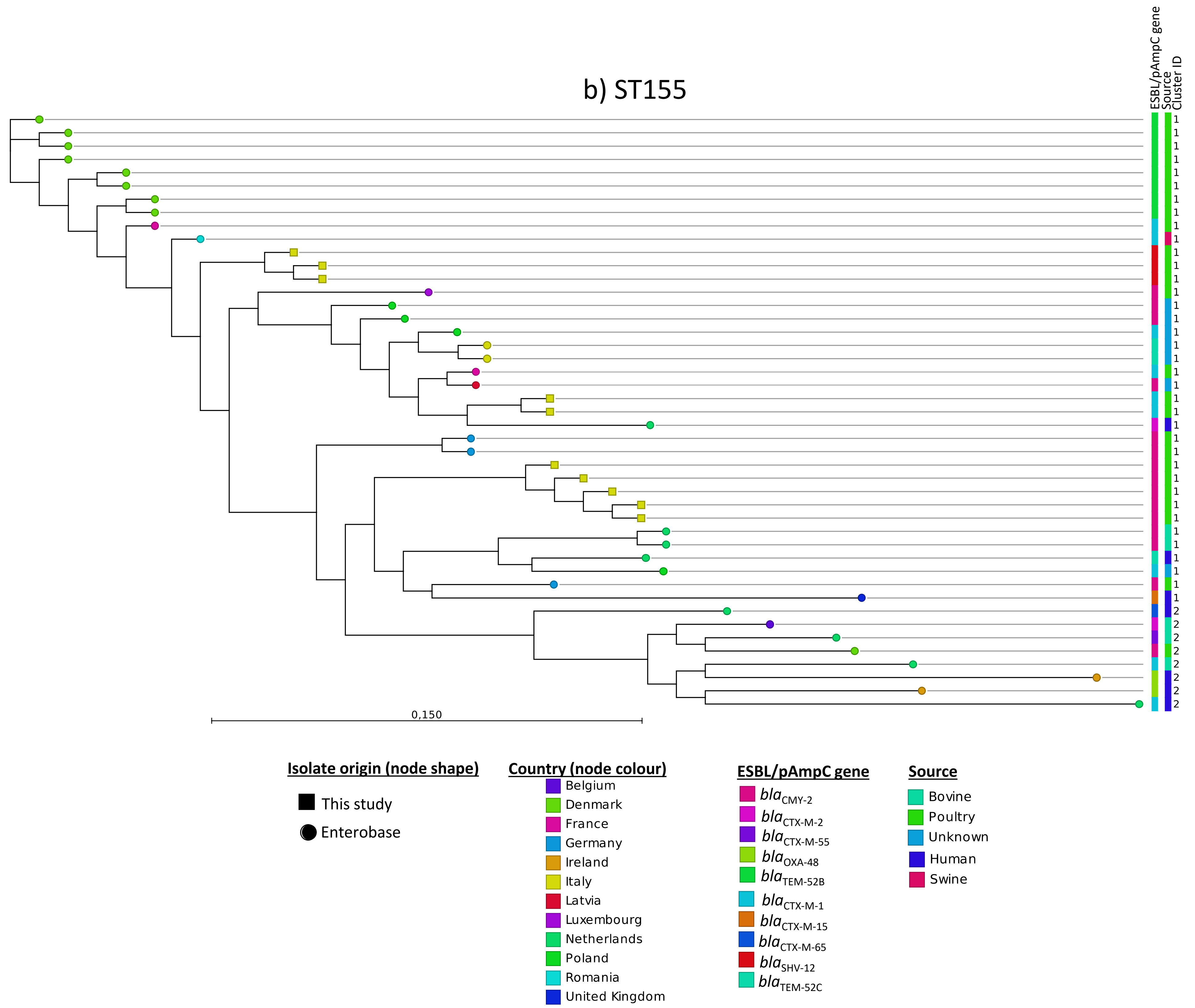

b) ST155
ESBL/pAmpC gene
Source
Cluster ID
0,150
Isolate origin (node shape)
This study
Enterobase
Country (node colour)
Belgium
Denmark
France
Germany
Ireland
Italy
Latvia
Luxembourg
Netherlands
Poland
Romania
United Kingdom
ESBL/pAmpC gene
blaCMY-2
blaCTX-M-2
blaCTX-M-55
blaOXA-48
blaTEM-52B
blaCTX-M-1
blaCTX-M-15
blaCTX-M-65
blaSHV-12
blaTEM-52C
Source
Bovine
Poultry
Unknown
Human
Swine

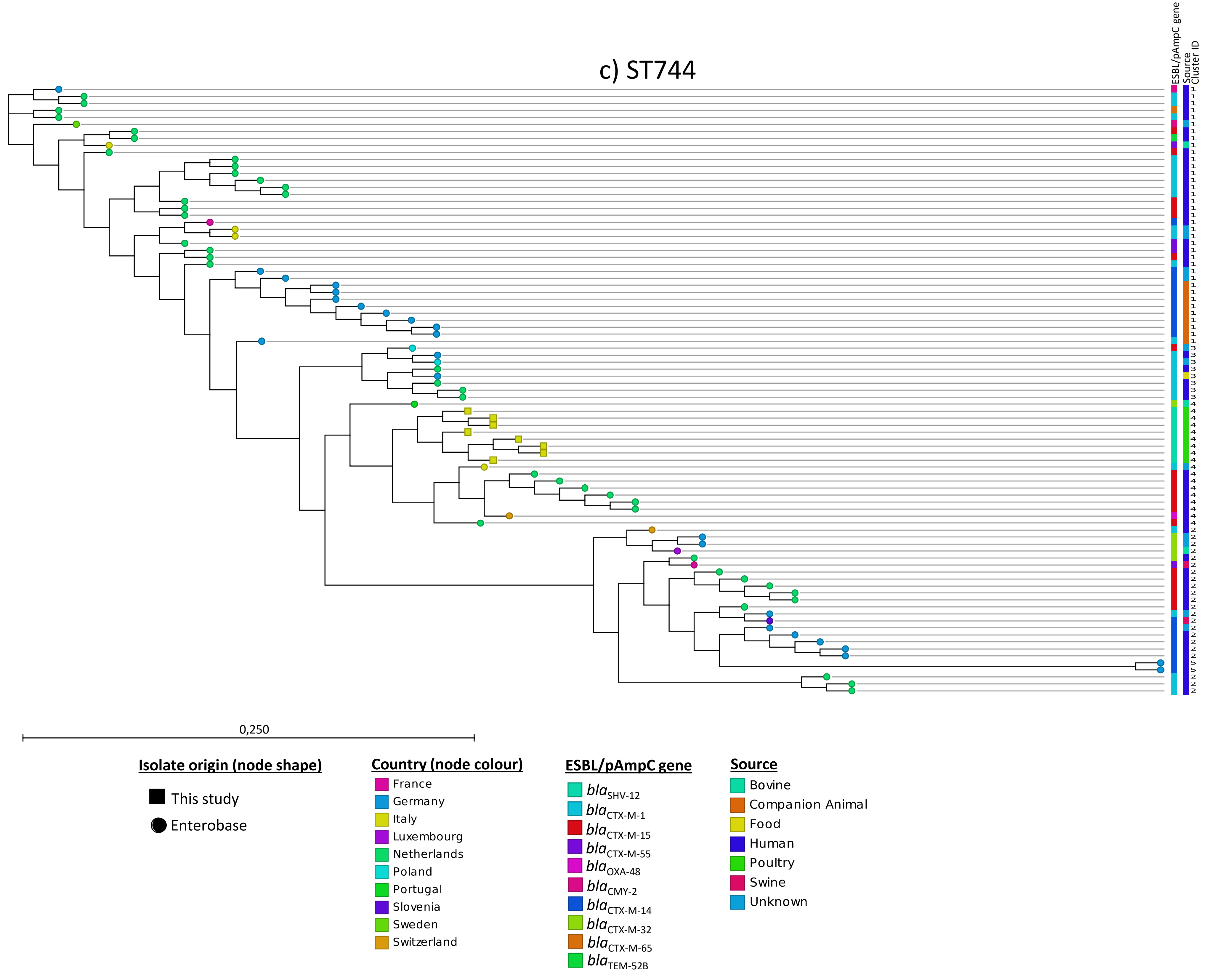

c) ST744
ESBL/pAmpC gene
Source
Cluster ID
0,250
Isolate origin (node shape)
This study
Enterobase
Country (node colour)
France
Germany
Italy
Luxembourg
Netherlands
Poland
Portugal
Slovenia
Sweden
Switzerland
ESBL/pAmpC gene
blaSHV-12
blaCTX-M-1
blaCTX-M-15
blaCTX-M-55
blaOXA-48
blaCMY-2
blaCTX-M-14
blaCTX-M-32
blaCTX-M-65
blaTEM-52B
Source
Bovine
Companion Animal
Food
Human
Poultry
Swine
Unknown

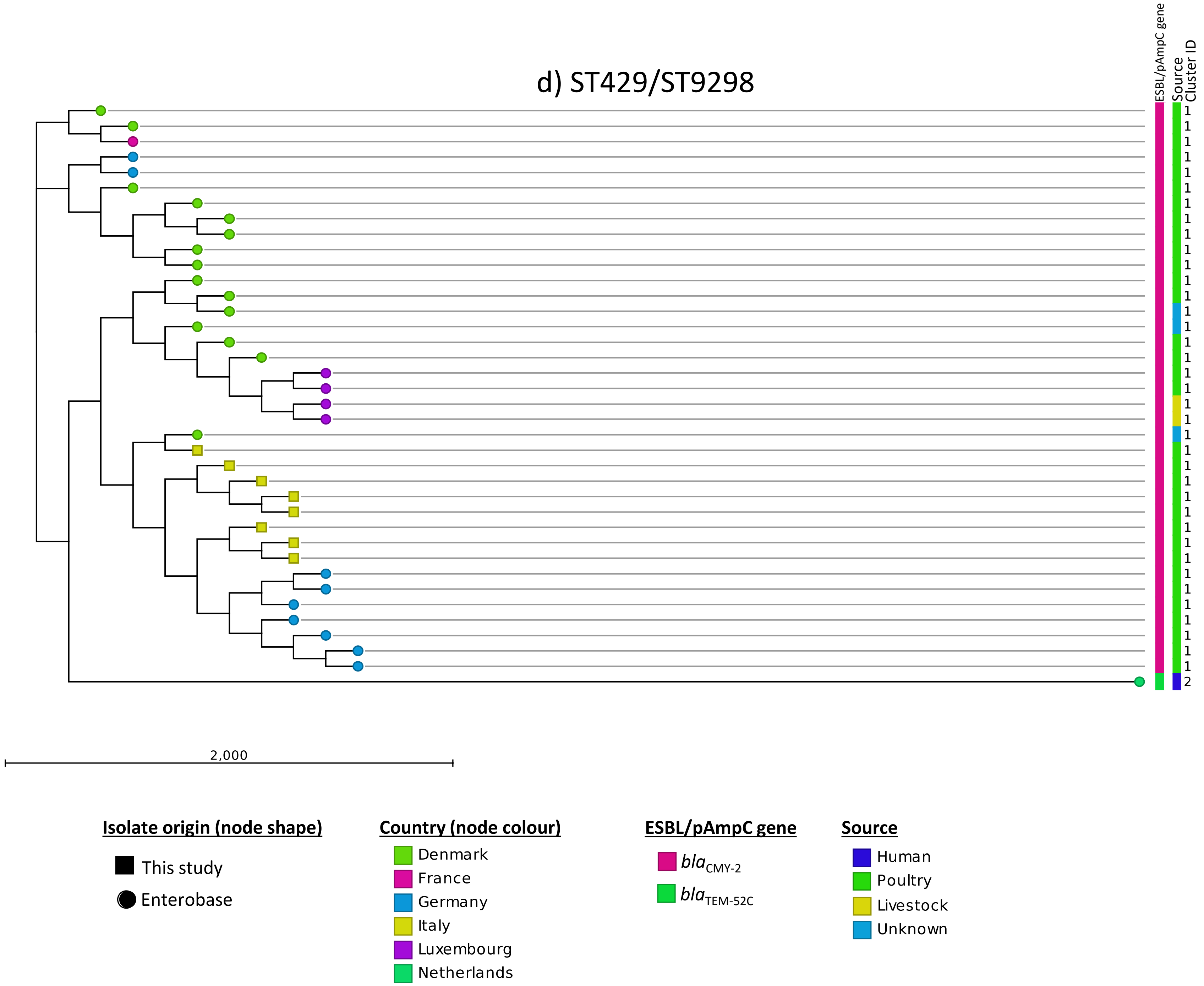

d) ST429/ST9298
ESBL/pAmpC gene
Source
Cluster ID
1
2
2,000
Isolate origin (node shape)
This study
Enterobase
Country (node colour)
Denmark
France
Germany
Italy
Luxembourg
Netherlands
ESBL/pAmpC gene
blaCMY-2
blaTEM-52C
Source
Human
Poultry
Livestock
Unknown

Supplementary Figure S2. Phylogenetic analysis of a) ST457, b) ST155, c) ST744, d) ST429/ST9298 ESBL/pAmpC-EC of this study and of various sources in Europe that were parsed from Enterobase database. SNP analysis was done with CSI phylogeny. The isolates of this study have a rectangular node shape while those from Enterobase are indicated with a circle. Source niche, ESBL/pAmpC genes, and country of origin are colour-coded according to the legend. Numbers next to ribbons indicate the cluster of isolates calculated with hierBAPS. Scale bar refers to the branch lengths, which are measured in the number of substitutions per site. Tree nodes are ordered after their increasing branch lengths.
